# Bifurcation Configuration Is an Independent Risk Factor for Aneurysm Rupture Irrespective of Location

**DOI:** 10.3389/fneur.2019.00844

**Published:** 2019-08-06

**Authors:** Qinglin Liu, Peng Jiang, Yuhua Jiang, Shaolin Li, Huijian Ge, Hengwei Jin, Youxiang Li

**Affiliations:** ^1^Department of Interventional Neuroradiology, Beijing Neurosurgical Institute, Beijing Tiantan Hospital, Capital Medical University, Beijing, China; ^2^Beijing Neurointerventional Engineering Center, Beijing, China

**Keywords:** intracranial aneurysms, risk assessment, bifurcation, sidewall, morphology

## Abstract

**Background:** Bifurcation and sidewall aneurysms have different rupture risks, but whether this difference comes from the location of the aneurysm is not clear. The objective of this study is to illustrate the rationality of ranking bifurcation configuration as an independent risk factor for aneurysm rupture.

**Methods:** Morphological features of 719 aneurysms (216 ruptured) were automatically extracted from a consecutive cohort of patients via *PyRadiomics*. Rupture risks and morphological features were compared between bifurcation and sidewall aneurysms, and lasso regression was applied to explore the morphological determinants for rupture in bifurcation and sidewall aneurysms. Rupture risks and morphological features of bifurcation aneurysms in different locations were analyzed. Multivariate regression was performed to explore the risk factors for aneurysm rupture.

**Results:** Twelve morphological features were automatically extracted from *PyRadiomics* implemented in Python. The rupture risks were higher in bifurcation aneurysms (*P* < 0.01), and morphological features Elongation and Flatness were much lower in ruptured bifurcation than sidewall aneurysms (*P* = 0.036, 0.011, respectively). Elongation and Flatness were the morphological determinants for rupture in bifurcation aneurysms, whereas Elongation and SphericalDisproportion were determinants for sidewall aneurysms. Different rupture risks and morphological features were found between sidewall and bifurcation aneurysms of the same location, and among bifurcation aneurysms of different locations. In multivariate regression, bifurcation configuration was an independent risk factor for aneurysm rupture (OR 3.007, 95% CI 1.752–5.248, *P* < 0.001).

**Conclusions:** Sidewall and bifurcation aneurysms and bifurcation aneurysms of different locations have different rupture risks and morphological features. Bifurcation configuration is an independent risk factor for aneurysm rupture irrespective of location.

## Introduction

There are several controversies for determining the optimal treatment strategy for an incidentally detected unruptured intracranial aneurysm. The high prevalence (3.2%, 95% CI 1.9–5.2%) and relative low rupture rate (0.25%) of intracranial aneurysms makes observation seems reasonable (1), but the catastrophic consequence once it ruptures (42% of overall 28-day fatality rate) (2) has pushed a majority of patients for prophylactic treatment (3). However, no treatment comes without risks, the overall morbidity and mortality rate 30 days after treatment in patients without previous hemorrhage is 13.7 and 9.7%, in open surgical and endovascular groups, respectively (4). All these controversies raised the issue of aneurysm rupture risk stratification.

Some patient and the aneurysm-related risk factors have been derived from large cohorts of patients and enrolled into the scoring system for predicting aneurysm rupture risks (5, 6). In the PHASES scoring system, the enrolled patient-related risk factors were population, hypertension, age, earlier hemorrhage from another aneurysm, and the aneurysm-related risk factors were size and site (6). The regularity of the aneurysm was also found to be closely related to aneurysm rupture risks and has been enrolled into the Japanese scoring system for predicting 3-year rupture risks (5). Regularity indexes, such as size ratio (SR), flow angle (FA), height/width ratio (H/W ratio), aspect ratio (AR), non-sphericity index (NSI), ellipticity index (EI), and undulation index (UI) were defined for delineating the morphology of the aneurysm and predicting its rupture risks (7–10). SR, FA, AR, and H/W ratio are easy to calculate in a 2-dimensional projection but always are semi-objective, as measurements of the same aneurysm from different projections may be different. Projections may be different between raters as the different comprehension of the aneurysm. Furthermore, SR, FA, and AR are measured in a 2-dimentional projection and are insufficient to delineate the overall morphology of a 3-dimentional object. EI, NSI, and EI are complex to calculate. Recently, Leemans et al. proposed an algorithm for automatically calculating these parameters for exploring morphological changes during aneurysm growth (11). In this study, the authors found these measurements are objective and repeatable while preserving a close connection to the current measurement methodology (11). More studies still were needed to illustrate the feasibility of these automatically calculated parameters for aneurysm rupture risk stratification. Furthermore, morphological parameters automatically extracted from *PyRadiomics* (Dana-Farber Cancer Institute, Brigham and Women's Hospital, Harvard Medical School) were calculated in a pixel-by-pixel manner, and could faithfully reflect the overall morphology of 3-dimensional object. The high consistency of these morphological parameters between raters makes them suitable for delineating the morphology of the aneurysm in rupture risk stratification (unpublished data).

A recent study has dichotomized aneurysms into sidewall and bifurcation subtypes, and these two types of aneurysms feature distinct morphological and hemodynamic properties (12). Bifurcation aneurysms are thought to have a higher rupture risk than sidewall ones (13). Furthermore, aneurysms of the basilar artery bifurcation and internal carotid artery bifurcation also have different rupture risks and hemodynamic properties (14). These results demonstrate that bifurcation configuration may be an independent risk factor for aneurysm rupture irrespective of aneurysm location.

To further illustrate our hypothesis, we compared the rupture risks and morphological features between bifurcation and sidewall aneurysms by locations, and among bifurcation aneurysms of different locations. Multivariate regression was performed to explore the risk factors for aneurysm rupture.

## Materials and Methods

### Patient Cohorts

The patients were enrolled from January 2015 to September 2018. The inclusion criteria were as follows: patients who had 3D digital subtraction angiography by Siemens Artis Zee System (Siemens Healthcare, Erlangen, Germany); a confirmed ruptured or unruptured diagnosis of aneurysm; sufficient image quality for 3D vessel construction with no artifacts to accurately represent aneurysm and parent vasculature; saccular aneurysm; and available clinical charts. Aneurysms combined with other vascular abnormalities such as arteriovenous malformation, arteriovenous fistula, and Moyamoya disease were excluded. A total of 719 aneurysms in 579 patients were enrolled in this study.

### Acquisition of Clinical and Morphological Features

This study included clinical variables that have been proven as risk predictors for aneurysm rupture. The variables were sex, hypertension, hyperlipemia, diabetes, smoking, and drinking status, and multiplicity and location of aneurysms (5, 6, 15–17). The clinical features of patients were collected from in-hospital medical records.

With regard to morphologic features, the vessels were reconstructed into 3D figures by using the Software 3D Slicer (version 4.8.0; http://www.slicer.org). Then, the aneurysm was manually segmented from the parent vessel by the aneurysm neck by two individual interventionists (Liu QL, Ge HJ). Subsequently, 12 morphological features were automatically extracted for each aneurysm via *PyRadiomics* platform implemented in Python (18, 19). Segmentation of the aneurysm and extraction of the morphological features from *Pyradiomics* were described in details previously (19). In brief, the sectional images of the vessel were imported to 3D slicer for vasculature reconstruction. Then, the aneurysm was segmented from the parent vessel, and the aneurysm and the parent vessel were exported into NRRD format files of the same size. Finally, the aneurysm and its corresponding parent vessel were read by the *Pyradiomics* program in python, the measurements of these morphological features were calculated in a pixel-by-pixel manner, and the results were exported and saved in an excel format file. The feature extraction progress was automatically executed by running a series of codes. The codes would be supplied to the readers from the corresponding author for any reasonable request.

These morphological features include the following: Compactness1 (a measure of how compact the shape is relative to a sphere), Compactness2 (a measure of how compact the shape is relative to a sphere), SurfaceArea (the total area of the shape), SurfaceVolumeRatio (the ratio of surface area to volume of a shape), Sphericity (a measure of the roundness of the shape relative to a sphere), SphericalDisproportion (the ratio of the surface area to the surface area of a sphere with the same volume), Maximum3DDiameter (the largest pairwise Euclidean distance between surface mesh vertices), Maximum2DDiameterSlice (the largest pairwise Euclidean distance between surface mesh vertices in the axial plane), Maximum2DDiameterColumn (the largest pairwise Euclidean distance between surface mesh vertices in coronal plane), Maximum2DDiameterrow (the largest pairwise Euclidean distance between surface mesh vertices in the sagittal plane), Elongation (a measure shows the relationship between the two largest principal components in the shape), and Flatness (a measure shows the relationship between the largest and smallest principal components in the shape). Detailed information of these features is available in the documentation for *PyRadiomics* (http://PyRadiomics.readthedocs.io/en/latest/).

### Definitions of Bifurcation and Sidewall Configurations

Discrimination of bifurcation and sidewall aneurysms has been reported (20). In brief, bifurcation aneurysms were defined as aneurysms which were located at a major bifurcation in the cerebral vasculature. Sidewall aneurysms were defined as aneurysms which originate from only one parent vessel or from the origin of a branch that was much smaller than the parent vessel (less than one-fifth the diameter). According to these definitions, anterior communicating artery aneurysms all belong to the bifurcation aneurysms, and posterior communicating artery aneurysms could either be grouped into bifurcation or sidewall aneurysms according to the relative diameter of the parent internal carotid artery and posterior communicating artery.

### Comparison of Rupture Risks and Morphological Features Between Sidewall and Bifurcation Aneurysms

All aneurysms were categorized into sidewall or bifurcation subtypes according to the definitions mentioned above. Rupture risks and morphological features were compared between all sidewall and bifurcation aneurysms, and aneurysms of the same location (the posterior communicating artery). Rupture risks and morphological features of ruptured bifurcation aneurysms at the anterior, and posterior communicating artery and the middle cerebral artery were compared.

### Morphological Determinants for Rupture in Bifurcation and Sidewall Aneurysms

Lasso regression was used for exploring the determinants for rupture in bifurcation, sidewall, and overall aneurysms. For each group, the two most important morphological determinants were selected, and the importance of the variates was compared. Prediction models for bifurcation aneurysms (model_BF), sidewall aneurysms (model_SW), and all aneurysms (model_all) were constructed with the selected morphological determinants in each group. Receiver operating curves (ROCs) were built and the performance of the models was compared by comparing areas under the curves (AUCs).

### Comparison of Rupture Risks and Morphological Features Between Bifurcation Aneurysms of Different Locations

Rupture risks and morphological features of ruptured bifurcation aneurysms at the anterior, and posterior communicating artery and the middle cerebral artery were compared. Firstly, the morphological differences between aneurysms of the three locations were compared as a whole. Secondly, if the difference of a morphological feature was significant, further comparison would be performed between aneurysms from each location.

### Multivariate Analysis for the Risk Factors for Aneurysm Rupture

Morphological features were standardized into a standard normal distribution. Morphological features, bifurcation configuration, and clinical features were enrolled for multivariate regression analysis, and backward stepwise regression was performed to explore the risk factors for aneurysm rupture. Odd ratio (OR) and 95% confidential interval (95% CI) of each variate were calculated. Variance inflation factors (VIFs) were calculated to test the collinearity of the variates.

### Statistical Analysis

All the statistical analysis was done with R (Version 3.5.2, R Foundation for Statistical Computing, Vienna, Austria). Comparison of the morphological features between two groups were conducted by Student *t*-test or Wilcox test, depending on the results from normality and variance equality test of each continuous variate. If the variates in the two groups fit normal distribution and their variance are equal, Student *t*-test would be adopted. Otherwise, Wilcox test would be adopted. Categorical variates between these groups were compared with chi-square test. Rupture rate between groups was compared with chi-square test. Morphological comparison of bifurcation aneurysms in different locations (the anterior communicating artery, the posterior communicating artery, and the middle cerebral artery) was performed with Kruskal Wallis test after the normality test of each subgroup, and Bonferroni test was used to paired comparison if the difference was significant (*P* <0.05) from the Kruskal Wallis test. The main packages used in this study were leaps, car, glmnet, DesTools, and pROC.

## Results

### Demographic Information of the Patients and Aneurysms

A total of 719 aneurysms in 579 patients were enrolled in this study. Three hundred and fifty-three aneurysms were defined as sidewall aneurysms (48 ruptured) and the rest 366 as bifurcation aneurysms (168 ruptured). The median size of the ruptured aneurysms was 6.276 mm, with no significant difference from the median size of unruptured aneurysms (6.010 mm, *P* = 0.231).

Clinical features of bifurcation and sidewall aneurysms were summarized in Table 1. As the table showed, female sex was more predominant in sidewall than bifurcation aneurysms (74.2 vs. 59.6%, *P* < 0.01), and hypertension, hyperlipemia, smoking, and drinking were more prevalent in bifurcation aneurysms (*P* < 0.05). Bifurcation or sidewall configuration was greatly related to aneurysm location (*P* < 0.01), most bifurcation aneurysms located at anterior cerebral artery, anterior, and posterior communicating artery, and the posterior circulation (69.9%), and middle cerebral artery (26.5%). Sidewall aneurysms mainly located at the internal carotid artery (75.6%). The detailed location distribution of aneurysms was demonstrated in Table 2. All anterior communicating artery aneurysms and 93.3% of the middle cerebral artery aneurysms were bifurcation type, and 95.4% (267/280) of the internal carotid artery aneurysms were sidewall type. Of the 121 posterior communicating aneurysms, 55 were sidewall type (14 ruptured), and 66 were bifurcation type (38 ruptured).

**Table 1:** Patients and aneurysm information.

	**Bifurcation (*N* = 366)**	**Sidewall (*N* = 353)**	***P***
Sex (female)	218 (59.6%)	262 (74.2%)	<0.001
Age (>65)	152 (41.5%)	124 (35.1%)	0.091
Hypertension	242 (66.1%)	150 (42.5%)	<0.001
Hyperlipemia	72 (19.7%)	23 (6.5%)	<0.001
Diabetes	49 (13.4%)	43 (12.2%)	0.800
Smoke	101 (27.6%)	58 (16.4%)	<0.001
Wine	95 (30.0%)	58 (16.4%)	0.002
Multiplicity	115 (31.4%)	147 (41.6%)	0.006
Location			<0.001
ACA/Acom/Pcom/posterior	256 (69.9%)	79 (22.4%)	
MCA	97 (26.5%)	7 (2.0%)	
ICA	13 (3.6%)	267 (75.6%)	

*ACA, anterior cerebral artery; Acom, anterior communicating artery; Pcom, posterior communicating artery; posterior, posterior circulation; MCA, middle cerebral artery; ICA, internal carotid artery*.

**Table 2:** Location distribution of the aneurysms.

**Location**	**Side wall**	**Bifurcation**	**Total (%)**
ACA	13	6	19 (2.6%)
Acom	0	140	140 (19.5%)
BA	2	30	32 (4.5%)
ICA	267	13	280 (38.9%)
MCA	7	97	104 (14.5%)
PCA	3	1	4 (0.6%)
Pcom	55	66	121 (16.8%)
PICA	2	1	3 (0.4%)
VA	4	11	15 (2.1%)

*BA, basilar artery; PCA, posterior cerebral artery; PICA, posterior inferior cerebellar artery; VA, vertebral artery; VA, vertebral artery*.

### Rupture Risk and Morphological Comparison Between Bifurcation and Sidewall Aneurysms

45.9% (168/366) of all bifurcation aneurysm was ruptured, which was significantly higher than sidewall aneurysms (13.6%, 48/353) (*P* < 0.001). 57.6% (38/66) bifurcation posterior communicating artery aneurysms were ruptured, and only 25.5% (14/55) sidewall ones were ruptured, with a significant difference between these two groups (*P* < 0.001). These results demonstrated that bifurcation aneurysms had a higher rupture risk than sidewall ones, even at the same location.

Elongation (*P* = 0.036) and Flatness (*P* = 0.011) were lower in ruptured bifurcation aneurysms than sidewall ones, demonstrating that bifurcation aneurysms were more irregular than sidewall aneurysms when rupture. Other morphological features showed no obvious difference.

To eliminate the effect of location on the morphology of ruptured aneurysms, bifurcation and sidewall aneurysms of the posterior communicating artery were enrolled for analysis. The result was demonstrated in Table 3. Maximum3DDiameter, Maximum2DDiameterColumn, Maximum2DDiameterRow, and SurfaceArea were larger in ruptured bifurcation posterior communicating artery aneurysms than sidewall ones (*P* = 0.023, 0.017, <0.001, and 0.037, respectively). These results implied that bifurcation posterior communicating artery aneurysms were larger than sidewall ones when they rupture. Regularity indexes did not show any differences between these two groups in our study (*P* > 0.05).

**Table 3:** Morphological comparison of posterior communicating artery aneurysms.

**Morphological features**	**Overall (*****N*** **= 121)**		***P***	**Ruptured (*****N*** **= 52)**		***P***
	**Side wall (*N* = 55) mean ± SD/median (IQR)**	**Bifurcation (*N* = 66) mean ± SD/median (IQR)**		**Side wall (*N* = 14) mean ± SD/median (IQR)**	**Bifurcation (*N* = 38) mean ± SD/median (IQR)**	
Compactness1	0.035 ± 0.004	0.035 ± 0.003	0.527	0.033 ± 0.004	0.034 ± 0.003	0.708
Compactness2	0.435 ± 0.086	0.443 ± 0.078	0.557	0.417 (0.372, 0.439)	0.410 (0.349, 0.435)	0.895
SurfaceArea	78.092 (42.071, 127.532)	90.169 (58.380, 130.071)	0.350	57.530 (33.940, 125.296)	103.305 (71.933, 164.665)	0.037
SurfaceVolumeRatio	1.855 (1.517, 2.629)	1.751 (1.456, 2.076)	0.241	2.273 (1.809, 2.765)	1.672 (1.369, 1.960)	0.018
Sphericity	0.754 ± 0.051	0.760 ± 0.046	0.518	0.733 ± 0.057	0.739 ± 0.047	0.699
SphericalDisproportion	1.332 (1.278, 1.360)	1.314 (1.254, 1.355)	0.389	1.338 (1.316, 1.390)	1.346 (1.320, 1.420)	0.895
Maximum3DDiameter	6.656 (4.436, 8.135)	7.226 (5.472, 8.519)	0.169	5.641 (4.375, 8.095)	8.193 (6.691, 9.504)	0.023
Maximum2DDiameterSlice	5.446 (4.203, 7.359)	6.230 (4.983, 7.345)	0.392	5.121 (3.596, 7.784)	6.835 (5.607, 7.825)	0.132
Maximum2DDiameterColumn	5.983 (4.315, 7.198)	5.945 (4.525, 7.330)	0.551	4.718 (4.220, 6.764)	6.897 (5.231, 8.633)	0.017
Maximum2DDiameterRow	5.471 (3.931, 7.297)	6.279 (4.631, 7.652)	0.097	4.374 (3.550, 7.146)	7.000 (5.302, 8.992)	<0.001
Elongation	0.740 (0.652, 0.816)	0.726 (0.594, 0.838)	0.627	0.691 (0.601, 0.835)	0.603 (0.560, 0.814)	0.301
Flatness	0.629 (0.535, 0.736)	0.571 (0.451, 0.690)	0.070	0.593 (0.478, 0.781)	0.467 (0.440, 0.671)	0.071

*SD, standard derivation; IQR, interquartile range*.

### Different Morphological Determinants for Rupture in Bifurcation and Sidewall Aneurysms

**Figure 1:**
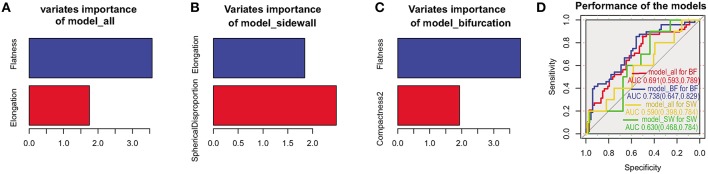
Morphological determinants for all, sidewall, and bifurcation aneurysms. Morphological determinants and importance of the variates in all **(A)**, sidewall **(B)**, and bifurcation **(C)** aneurysms. Performances of model_all, model_BF, model_SW in predicting the rupture status of bifurcation and sidewall aneurysms **(D)**.

Morphological determinants for rupture in bifurcation, sidewall, and all aneurysms were detected with lasso regression. As Figure 1 shown, the morphological determinants of sidewall aneurysms were Elongation and SphericalDisproportion, while Flatness and Compactness2 were detected as the morphological determinants for bifurcation aneurysms. The morphological determinants for all aneurysms were Flatness and Elongation. To further elucidate the rationality of these different morphological determinants for bifurcation and sidewall aneurysms, performances of the models constructed with their own determinants were compared. As Figure 1D showed, for predicting the rupture status of bifurcation aneurysms, the AUC of model_BF was significantly higher than that of model_all (0.738 vs. 0.691, *P* = 0.045). The similar result was got between model_SW and model_all in predicting the rupture status for sidewall aneurysms (0.626 vs. 0.589, *P* = 0.049).

### Rupture Risks and Morphological Analysis for Bifurcation Aneurysms of Different Locations

The rupture rate of bifurcation aneurysms at the anterior, and posterior communicating artery and middle cerebral artery aneurysm was 57.1% (80/140), 57.6% (38/66), and 28.9% (28/97), respectively, with significant differences among these three groups (*P* < 0.001). Significant differences were found between rupture rate of the anterior communicating artery and middle cerebral artery bifurcation aneurysms (57.1 vs. 28.9%, *P* < 0.001), and between the posterior communicating artery and the middle cerebral artery bifurcation aneurysms (57.6 vs. 28.9%, *P* < 0.001). No difference was found between the anterior and posterior communicating artery groups (57.1 vs. 57.6%, *P* = 1.00). These results demonstrate that location may be an independent risk factor for aneurysm rupture irrespective bifurcation configuration.

Morphology of bifurcation aneurysms in anterior, and posterior communicating artery and middle cerebral artery were compared and the results were summarized in Table 4. Except for Elongation and Flatness, all the other morphological features of ruptured bifurcation aneurysms were significantly different among these three locations. Paired morphological analysis between locations showed that the morphological differences all came from posterior communicating artery aneurysms and aneurysms of the other two locations (*P* < 0.05). Morphology was similar between bifurcation aneurysms at the anterior communicating artery and middle cerebral artery (*P* > 0.05). These results demonstrate that location may influence the morphology of aneurysm when it ruptures irrespective of bifurcation configuration.

**Table 4:** Morphological comparison of ruptured bifurcation aneurysms of different locations.

**Morphological features**	**All ruptured aneurysms (*****N*** **= 146)**			**Overall**	***P*****-value**		
	**Acom (*N* = 80) mean ± SD/median (IQR)**	**MCA (*N* = 28) mean ± SD/median (IQR)**	**Pcom (*N* = 38) mean ± SD/median (IQR)**		**Acom-MCA**	**Acom-Pcom**	**MCA-Pcom**
Compactness1	0.036 (0.034, 0.038)	0.037 (0.036, 0.039)	0.034 (0.031, 0.035)	<0.001	0.380	0.013	<0.001
Compactness2	0.463 (0.402, 0.522)	0.494 (0.452, 0.528)	0.410 (0.349, 0.435)	<0.001	0.380	0.013	<0.001
SurfaceArea	59.602 (38.464, 98.622)	51.274 (35.582, 101.413)	103.305 (71.933, 164.665)	<0.001	1.000	<0.001	0.002
SurfaceVolumeRatio	2.058 (1.667, 2.415)	2.094 (1.634, 2.737)	1.672 (1.369, 1.960)	0.003	1.000	0.004	0.027
Sphericity	0.773 (0.738, 0.805)	0.791 (0.768, 0.808)	0.743 (0.704, 0.758)	<0.001	0.380	0.013	<0.001
SphericalDisproportion	1.293 (1.242, 1.354)	1.265 (1.237, 1.302)	1.346 (1.320, 1.420)	<0.001	0.380	0.013	<0.001
Maximum3DDiameter	6.055 (4.528, 7.552)	5.370 (4.557, 7.571)	8.193 (6.691, 9.504)	<0.001	1.000	<0.001	0.001
Maximum2DDiameterSlice	4.609 (3.763, 6.514)	4.564 (3.695, 6.255)	6.835 (5.607, 7.825)	<0.001	1.000	<0.001	0.002
Maximum2DDiameterColumn	4.976 (4.088, 5.937)	4.797 (3.989, 6.004)	6.898 (5.231, 8.633)	<0.001	1.000	0.001	0.001
Maximum2DDiameterRow	5.103 (3.954, 6.959)	5.083 (3.932, 6.158)	7.000 (5.302, 8.992)	<0.001	1.000	<0.001	0.004
Elongation	0.681 (0.546, 0.765)	0.701 (0.641, 0.771)	0.604 (0.560, 0.814)	0.367			
Flatness	0.563 (0.444, 0.617)	0.588 (0.510, 0.663)	0.467 (0.440, 0.671)	0.066			

### Bifurcation Configuration Is an Independent Risk Factor for Aneurysm Rupture

When bifurcation configuration was enrolled for predicting aneurysm rupture with other morphological and clinical features, we found that bifurcation configuration was an independent risk factor (OR 3.007, 95% CI 1.752–5.248; Table 5). Other risk factors included surfaceVolumeRatio (OR 1.492, 95% CI 1.213–1.841), hyperlipemia (OR 2.550, 95% CI 1.491–4.399), and smoking (OR 2.232, 95% CI 1.115–4.484). Compactness2 (OR 0.598, 95% CI 0.470–0.765), Elongation (OR 0.689, 95% CI 0.544–0.868), multiplicity (OR 0.369, 95% CI 0.234–0.574), male sex (OR 0.389, 95% CI 0.208–0.706), location at middle cerebral artery (OR 0.539, 95% CI 0.310–0.924) and internal carotid artery (OR 0.286, 95% CI 0.150–0.541) were protective factors for aneurysm rupture (Table 5). As shown by the VIFs in Table 5, no collinearity was found between these enrolled variates.

**Table 5:** Multivariate analysis for determining the risk factors for aneurysm rupture.

**Risk factor**	**Coefficient**	**OR**	**95% CI**	***P***	**VIF**
Compactness2	−0.514	0.598	(0.470, 0.756)	<0.001	1.407
SurfaceVolumeRatio	0.400	1.492	(1.213, 1.841)	<0.001	1.126
Elongation	−0.373	0.689	(0.544, 0.868)	0.002	1.366
Hyperlipemia	0.936	2.550	(1.491, 4.399)	<0.001	1.035
Wine (yes)	0.545	1.725	(0.836, 3.546)	0.138	2.481
Smoke (yes)	0.803	2.232	(1.115, 4.484)	0.023	2.410
Multiple (yes)	−0.996	0.369	(0.234, 0.574)	<0.001	1.093
Sex (male)	−0.943	0.389	(0.208, 0.706)	0.002	2.174
Age (>65)	−0.388	0.678	(0.447, 1.024)	0.066	1.045
Bifurcation	1.101	3.007	(1.752, 5.248)	<0.001	1.715
Location at MCA	−0.617	0.539	(0.310, 0.924)	0.026	1.724
Location at ICA	−1.252	0.286	(0.150, 0.541)	<0.001	

## Discussion

Detection of unruptured intracranial aneurysms is increasing, but identifying the dangerous ones poses a great challenge. According to the international study of unruptured intracranial aneurysms (ISUIA), aneurysms that <10 mm showed a 5-year rupture risk of 0.05% (4), which could be negligible. But in retrospective studies, a great proportion of ruptured aneurysms were small aneurysms. In Forget's study, 85.6% of all ruptured aneurysms were smaller than 10 mm (21). In our series, the median maximal diameter of ruptured aneurysms was 6.276 mm, and the proportion of ruptured aneurysm smaller than 5, 7, and 10 mm was 31.9% (69/216), 59.7% (129/216), and 89.8% (194/216), respectively. These results demonstrate that size is far from sufficient for predicting aneurysms rupture risks.

Irregularity of the aneurysm was found an independent risk factor for aneurysm rupture irrespective of size (22), and the existence of daughter sacs was enrolled into the Japanese scoring system (5). Complex morphological indexes that have been proposed to delineate the regularity of aneurysms include SR, FA, AR, H/W, EI, and UI (7–10). Besides, we have introduced *PyRadiomics* derived morphological features in aneurysm risk stratification and the results were satisfying (our unpublished data). These morphological parameters could reflect the regularity of the aneurysm in a quantitative manner.

To test the rationality of discriminating bifurcation and sidewall configurations for aneurysm rupture risk stratification, we explored the morphological rupture determinants for bifurcation and sidewall aneurysms, respectively. We found that Elongation and SphericalDisproportion were morphological determinants for rupture in sidewall aneurysms, while Flatness and Compactness2 were morphological determinants for rupture in bifurcation aneurysms. This difference may imply that bifurcation and sidewall aneurysms may have different rupture mechanism. The better prediction performance of both the models for bifurcation aneurysms (0.738 vs. 0.691, *P* = 0.045) and sidewalls aneurysm (0.626 vs. 0.589, *P* = 0.049) than the overall aneurysms further rationalized the discrimination of bifurcation and sidewall aneurysms in aneurysm rupture stratification. The similar conclusion was also proposed previously (20).

Location of the aneurysm was another risk factor for determining its rupture status. In ISUIA, aneurysms located in the posterior circulation and posterior communicating artery have a higher rupture risk than other locations, irrespective the size of the aneurysm (4). Both in the PHASES and the Japanese scoring systems, the location was enrolled as an independent risk factor for predicting aneurysm rupture (5, 6). Other patient-related risk factors such as smoking, drinking, hypertension, age, sex, and populations have also been fully elucidated (5, 6, 15–17).

Recent studies have revealed that bifurcation aneurysms had a higher rupture risk than sidewall ones (13), and bifurcation and sidewall aneurysm had different hemodynamic features, which was thought to be closely related with aneurysm rupture (12). As bifurcation or sidewall configuration greatly correlates with the location of the aneurysm (*P* < 0.001; Table 1), whether the higher rupture risks of bifurcation aneurysms than sidewall ones (3) comes from the differences of their location was not clear. In our study, the rupture rate of bifurcation aneurysms was 45.9%, which was significantly higher than that of sidewall aneurysms (13.6%, *P* < 0.001). Bifurcation aneurysms mainly located at anterior communicating artery, middle cerebral artery, basilar tip, and the posterior communicating artery (91.0%, 333/366), while sidewall aneurysms mainly located at the internal carotid artery and the posterior communicating artery (91.2%, 322/353). To test whether the differences comes from their location differences, rupture risks of these two types of aneurysms at the same location (posterior communicating artery) were compared. The rupture rate of bifurcation posterior communicating artery aneurysms was significantly higher than sidewall ones (74.5 vs. 25.5%, *P* < 0.001), demonstrating bifurcation configuration might be an independent risk factor for aneurysm rupture irrespective of location. To further validate this conclusion, the morphology of these two types of aneurysms were compared. Interestingly, we found that ruptured bifurcation aneurysms of this site were much larger than sidewall ones (Table 3). It means that a bifurcation posterior communicating aneurysm may grow larger before rupture than the sidewall ones, and we can infer that in posterior communicating aneurysms of the same size, bifurcation ones should be safer, this was contrast to the fact that bifurcation aneurysms feature a higher rupture risk. This result further supported the conclusion that bifurcation configuration was a risk factor independent of location.

Doddasomayajula et al. have reported bifurcation aneurysms of the anterior and posterior circulation have different rupture risks and hemodynamic features (14). But whether these differences also exist among different locations of anterior circulation was unclear. In this study, ruptures risks and morphology of bifurcation aneurysms of different locations in the anterior circulation were compared. Both bifurcation aneurysms of the anterior and posterior communicating artery have a higher rupture risks than those at the middle cerebral artery (57.1 vs. 28.9%, *P* < 0.001; 57.6 vs. 28.9%, *P* < 0.001, respectively), and the rupture risk of bifurcation aneurysms at anterior and posterior communicating artery was similar (57.1 vs. 57.6%, *P* = 1.00). As to morphological analysis, ruptured bifurcation aneurysms at the posterior communicating artery were larger and more irregular than those at the anterior communicating artery and the middle cerebral artery (*P* < 0.05), and similar morphology and size were found between anterior communicating artery and middle cerebral artery bifurcation aneurysms (*P* > 0.05; Table 4). Lager diameters of the posterior communicating artery bifurcation aneurysms may be related to the larger parent artery. These results demonstrate that location is independent of bifurcation configuration and morphology in predicting aneurysm rupture.

To exclude the confusing effect from other risk factors, we took these well-defined risk factors as well as the bifurcation configuration into a multivariate logistic regression and found that bifurcation configuration was an independent risk factor for predicting aneurysm rupture factor (OR 3.007, 95% CI 1.752–5.248; Table 5). The similar result was reported by other authors (13). In their study, the location of the aneurysm was simply dichotomized into posterior circulation and others (13). In our study, location discrimination was the same as those described in the PHASES scoring system (6). Other risk factors in our study, such as sex, smoking, location, and irregularity indexes were in accordance with other studies (1, 3, 6). As the disappointing performance of PHASES scoring system in retrospective studies (23), we recommend bifurcation configuration could be considered as a supplement when evaluating the rupture risks of an unruptured aneurysm with the PHASES scoring system. The weight of this factor needs further investigation.

## Limitations

There are some limitations in our study. First, all patients in this study were from a single center with the same population. Second, other morphology parameters such as SR, AR, and FA et al. were not enrolled for analysis as the variance between raters, but they are valuable in predicting aneurysm rupture. Third, this is a retrospective study, patient selection bias is inevitable as most of the aneurysms enrolled in this study were indeed candidates for preventive treatment. Finally, although the total number of aneurysms is large, the volume of each subgroup is relatively small. Only aneurysms at the posterior communicating artery were enrolled for comparing the rupture risks and morphological features between sidewall and bifurcation subgroups. Large cohorts in multiple centers were needed to further elucidate this issue.

## Conclusions

Although some limitations, some conclusions still can be drawn from this study. Rupture risks vary between sidewall and bifurcation aneurysms, and among bifurcation aneurysms of different locations. Ruptured bifurcation aneurysms are larger and more irregular at posterior communicating artery than those at the anterior communicating artery and the middle cerebral artery. Although closely related to location, bifurcation configuration is an independent risk factor for aneurysm rupture irrespective of location.

## Data Availability

The datasets generated for this study are available on request to the corresponding author.

## Ethics Statement

This study was carried out following the rules of the Declaration of Helsinki, and was approved by the institutional Ethics Committee of Tiantan Hospital (2018-0114/2018-09-06). Written informed consent was obtained from each patient before operation or, for patients younger than 16, from their parents.

## Author Contributions

YL and QL made substantial contributions to the conception and design of the work. QL also contributed to the analysis of the data and writing of the manuscript. PJ, YJ, SL, HG, and HJ significantly contributed to the acquisition and interpretation of collected data.

### Conflict of Interest Statement

The authors declare that the research was conducted in the absence of any commercial or financial relationships that could be construed as a potential conflict of interest.
